# Interplay Between HGF/SF–Met-Ras Signaling, Tumor Metabolism and Blood Flow as a Potential Target for Breast Cancer Therapy

**DOI:** 10.18632/oncoscience.6

**Published:** 2013-12-11

**Authors:** Sari Natan, Galia Tsarfaty, Judith Horev, Roni Haklai, Yoel Kloog, Ilan Tsarfaty

**Affiliations:** ^1^ Department of Clinical Microbiology and Immunology, Sackler School of Medicine, Tel Aviv University; ^2^ Department of Diagnostic Imaging, Chaim Sheba Medical Center, Ramat Gan, Israel; ^3^ Department of Neurobiology, The George S. Wise Faculty of Life Sciences, Tel Aviv University, Tel Aviv, Israel; ^4^ This work was done in partial fulfillment of the requirements for the Ph.D. degree of S.N.

**Keywords:** HGF/Met/Ras as targets for therapy, Tumor metabolism, Functional molecular imaging

## Abstract

High glucose uptake and increase blood flow is a characteristic of most metastatic tumors. Activation of Ras signaling increases glycolytic flux into lactate, de novo nucleic acid synthesis and uncoupling of ATP synthase from the proton gradient. Met tyrosine kinase receptor signaling upon activation by its ligand, hepatocyte growth factor/scatter factor (HGF/SF), increases glycolysis, oxidative phosporylation, oxygen consumption, and tumor blood volume. Ras is a key factor in Met signaling. Using the Ras inhibitor S-trans,trans-farnesylthiosalicylic acid (FTS), we investigated interplay between HGF/SF-Met–Ras signaling, metabolism, and tumor blood-flow regulation.

In vitro, HGF/SF-activated Met increased Ras activity, Erk phosphorylation, cell motility and glucose uptake, but did not affect ATP. FTS inhibited basal and HGF/SF-induced signaling and cell motility, while further increasing glucose uptake and inhibiting ATP production. In vivo, HGF/SF rapidly increased tumor blood volume. FTS did not affect basal blood-flow but abolished the HGF/SF effect.

Our results further demonstrate the complex interplay between growth-factor-receptor signaling and cellular and tumor metabolism, as reflected in blood flow. Inhibition of Ras signaling does not affect glucose consumption or basal tumor blood flow but dramatically decreases ATP synthesis and the HGF/SF induced increase in tumor blood volume. These findings demonstrate that the HGF/SF-Met–Ras pathway critically influences tumor-cell metabolism and tumor blood-flow regulation. This pathway could potentially be used to individualize tumor therapy based on functional molecular imaging, and for combined signaling/anti-metabolic targeted therapy.

## INTRODUCTION

Tumors are characterized by an increase in oncogenic signaling, heightened metabolism, proliferation of antigenic blood vessels, and irregular hemodynamics [1]. Several researchers have demonstrated a mismatch between the metabolic demands of a tumor and its blood flow [2]. Met is the tyrosine kinase receptor for hepatocyte growth factor/scatter factor (HGF/SF). Activation of Met signaling pathways promotes cell proliferation, motility, survival, motility, invasion and angiogenesis [3, 4]. Aberrant expression of Met or HGF/SF or both was found to correlate with metastasis development and poor prognosis [5-8]. As a result, various anti-Met targeted compounds are currently under scrutiny as potential anti-cancer drugs, and some have reached successful phase III clinical trials [3, 9, 10].

Met has been shown to regulate tumor metabolism. Activation of Met leads to increased oxygen and glucose demand [11], causing augmented hypoxia. The hypoxia in turn induces Met expression and activation [12], resulting in a positive feedback loop of Met signaling. Activation of Met by HGF/SF leads to hemodynamic changes, expressed in a dramatic increase in blood flow to the tumor [13]. By exploiting this phenomenon, we developed a method for functional imaging of Met activation *in vivo* by using functional magnetic resonance imaging (fMRI) and contrast media ultrasound imaging (US-CMI) [13, 14].

Ras is known to be an essential downstream component of Met-HGF/SF signaling [15]. Oncogenic K-Ras plays a role in the metabolic reprogramming of cancer cells [16, 17]. Approximately 33% of all human tumors contain activating mutations in RAS oncogenes. K-Ras is mutated in 50%colon carcinoma, 30% in lung and 90% in pancreatic cancer [18]. Mutations of Ras and Raf, taken together with frequent hyperactivation of upstream receptor tyrosine kinases such as epidermal growth factor receptor in lung cancer [19] or Met in cancers of the stomach, kidney and liver [20], suggest that excessive signaling through the RTK-Ras-Raf-MEK-ERK pathway can induce proliferation in many tumors [18].

Ras signaling plays an important role not only in tumor cell growth but also in tumor metabolism. Ras signaling was recently shown to lead to metabolic reprogramming of tumor cells, resulting in increased metabolism through the nonoxidative pentose phosphate pathway, enabling: (1) increased NADPH for macromolecule biosynthesis, (2) reactive oxygen species detoxification and (3) ribose 5-phosphate for DNA/RNA synthesis [17, 21]. Ras inhibition was also shown to increase glucose uptake and to induce insulin sensitivity in myotubes, liver, fat and muscle[22].

S-trans,trans-farnesylthiosalicylic acid (FTS), also known as Salirasib, is a synthetic small molecule that acts as a potent Ras inhibitor [23-25], preventing activation of the Raf/MEK/ERK and the PI3-K/Akt pathways [23, 26, 27]. FTS inhibits the Ras/PI3-K pathway in a variety of tumor cells [27-30], and has also been shown to inhibit cell motility [29]. Moreover, FTS inhibits the *in-vivo* growth of a number of different tumors, including pancreatic cancer [31] [32], glioblastoma [33], and breast cancer [34].

Here we investigated the interplay between HGF/SF-Met and Ras signaling in the tumor metabolism and motility of a breast cancer cell line *in vitro* as well as on tumor growth and blood flow *in vivo*. We showed that FTS inhibits the increase in tumor blood flow induced by HGF/SF-Met activation. However, FTS also induced an increase in glucose consumption and a concomitant decrease in ATP. Our results thus demonstrated a mismatch between metabolism and blood flow in these tumors.

## RESULTS

### Inhibition of HGF/SF-induced ERK-Ras signaling phosphorylation by FTS

**Fig. 1:**
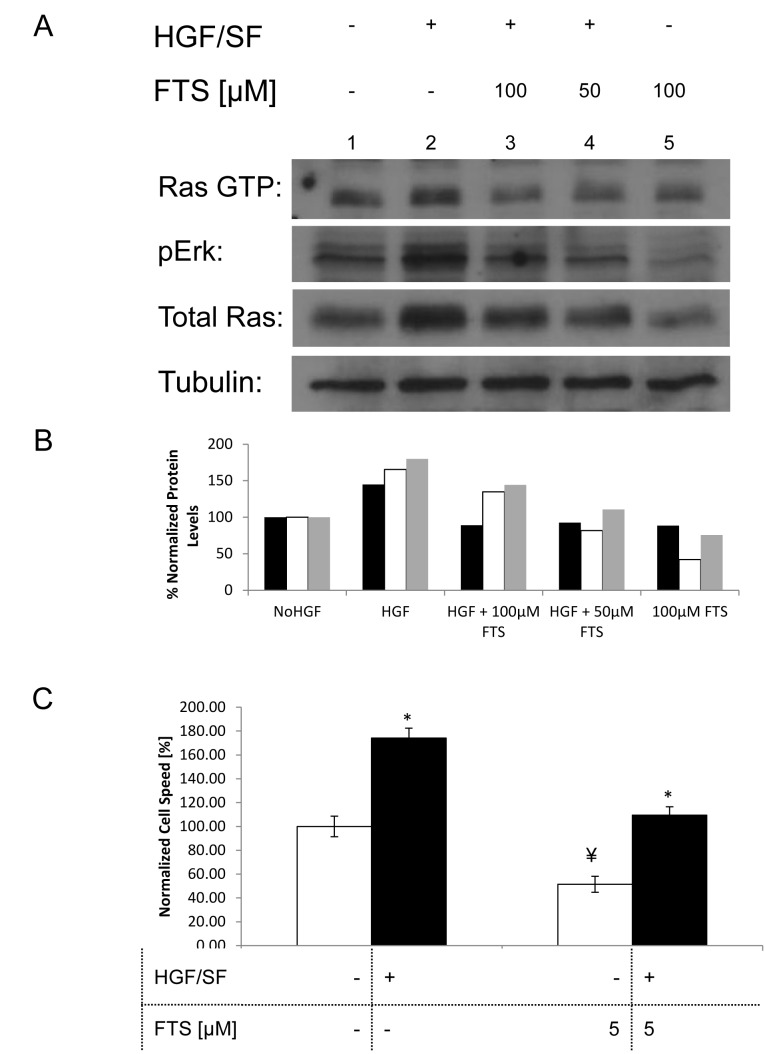
Effect of Ras inhibition on HGF/SF-induced Ras-ERK signaling and cell motility (A), DA3 cells were treated with vehicle (DMSO 0.1% control) or with HGF/SF (80ng/mL), 50 μM FTS+HGF/SF, 100 μM FTS +HGF/SF or 100 μM FTS. After 24h, the cells were lysed and the lysates were subjected to immunoblotting. These results demonstrate that HGF/SF activates Met downstream signaling via the ERK and Ras pathways, while FTS inhibits both basal level signaling and HGF/SF induced signaling via Ras and ERK. The bottom tubulin panel was used as the loading control. (B), Histogram of the protein expression levels, normalized to tubulin. Black bars represents the intensity of Ras GTP in the different samples, white bars represent pERK and gray Bars represent total Ras. (C), a scratch assay was performed by growing DA3 cells to confluency and then scratching the monolayer. Cell media was then exchanged to serum starvation media containing the different treatments. The cells were treated as following: vehicle (DMSO 0.1% control) or with HGF/SF (80ng/mL), 5 μM FTS, 5 μM FTS +HGF/SF. Cell motility rates are compared in the graph that demonstrates that HGF/SF activation of Met increases cell motility (n = 14, P < 0.02 represented by *), while its downstream inhibition by FTS inhibits cell motility (n = 15, P = 0.00025, represented **). Combined treatment with both HGF/SF and FTS leads to reduced cell motility as compared to vehicle treated cells (n = 12, P < 0.02).

To verify the inhibitory effects of FTS on HGF/SF-induced Ras activation in our DA3 model system, we examined active Ras (Ras-GTP), ERK phosphorylation and total Ras, by Western blot analysis (WB). DA3 cells were subjected for 24 hours to treatments with or without HGF/SF (80 ng/mL) and FTS (100 or 50 μM) (Fig. 1A, B). Compared to the control (vehicle alone – 0.1% DMSO) (basal level)} (lane 1), cells treated with HGF/SF alone (lane 2) showed an increase in Ras-GTP, in pERK, and in total Ras. This increase was inhibited when FTS (100 μM or 50 μM) was added to HGF/SF (lanes 3 and 4)

Compared to control cells (lane 1), cells treated with FTS (100 μM) without HGF/SF (lane 5) exhibited a small decrease in pERK. Quantitation of the Western analysis is presented in Fig. 1B. These results showed that HGF/SF increases Ras expression level (total Ras), Ras activation (Ras-GTP), and pERK, and that these effects are inhibited by FTS. Thus, FTS at concentrations of 50 μM to 100 μM inhibited HGF/SF-induced Ras-ERK signaling.

### Inhibition of HGF/SF-Induced Cell Motility by FTS

Using a wound-healing assay to examine the effect of HGF/SF and Ras on the motility of DA3 cells, we different treatments on the cell motility as depicted in the rate of cell motility calculated from the wound healing assay (Fig. 1C). Relative to untreated cells, the cells treated with HGF/SF alone closed the wound 75% faster (n = 14, P < 0.001), while the rate of wound closure with FTS treatment alone was decreased by 50% (n = 15, P < 0.001). The motility of cells that received combined treatment with HGF/SF and FTS did not differ significantly from that of untreated cells. These results showed that FTS inhibits both basal and HGF/SF-induced cell motility *in vitro*.

### Effects of HGF/SF and Ras inhibition on glucose uptake and ATP synthesis

**Fig. 2:**
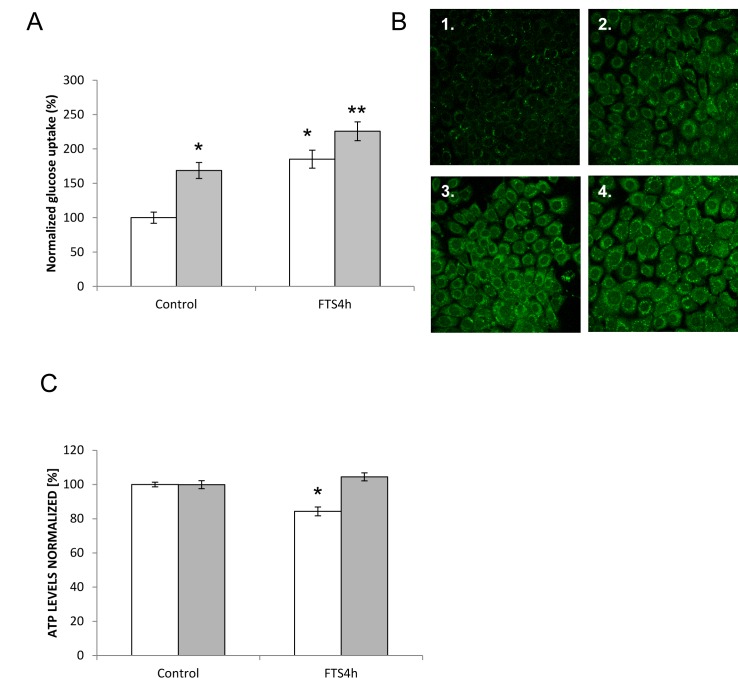
Effect of Ras inhibition on HGF/SF-induced glucose uptake and on ATP synthesis in tumor cells *in vitro* DA3 cells treated with vehicle (DMSO 0.1% control) or with HGF/SF (80ng/mL), 5 μM FTS, 5 μM FTS +HGF/SF.HGF/SF. (A), the cells were then incubated with the fluorescent glucose analog 2NDBG. Fluorescence was measured by confocal laser scanning microscopy (CLSM) and quantified by ImageJ. HGF/SF and FTS significantly increased the uptake of glucose separately and also in combination (*,P < 0.0001). (B), representative confocal images of DA3 cells treated with: 1. vehicle alone, 2. HGF/SF, 3. FTS, 4.HGF/SF and FTS. (C), despite the increase in glucose uptake, HGF/SF did not increase ATP production relative to baseline in control (vehicle-untreated) cells, yet FTS decreased ATP levels significantly (*, P<0.0001) and HGF/SF negated this effect (as seen in cells treated with both HGF/SF and FTS) (**, P < 0.0001).

To gain further knowledge of the effects of HGF/SF and Ras inhibition by FTS on tumor cell metabolism, we measured glucose uptake and ATP synthesis in cultured DA3 cells. The cells (starved of serum) were treated with HGF/SF (80 ng/mL for 24 hours) and/or FTS (5 μM for 4 hours) (Fig. 2A). Glucose uptake was increased by all 3 treatments compared to the control: the increase was 69% with HGF/SF alone, 85% with FTS alone (P < 0.0001), and 126% with combined treatment with HGF/SF and FTS (P = 0.03). In contrast to glucose uptake ATP levels were not affected by treatment with HGF/SF, either alone or in combination with FTS, while treatment with FTS alone decreased ATP levels by 24% (P < 0.0001) (Fig. 2B). These results demonstrated the uncoupling in these tumor cells between the effects FTS on glucose uptake and on ATP.

**Fig. 3:**
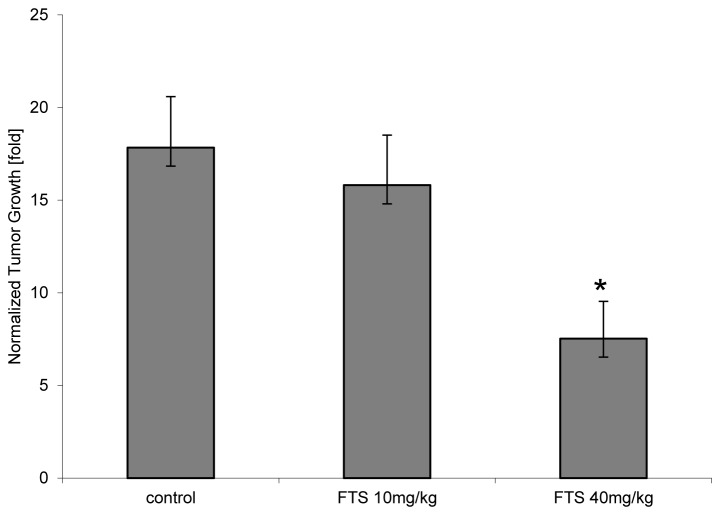
Effect of RAS inhibition on tumor growth Mice bearing DA3-xenograft mammary tumors were fed and treated as described in Materials and Methods. Tumors were measured by ultrasound, and their average growth at the end of the 3-week period of the experiment is presented as a bar chart. Compared to baseline, vehicle-treated tumors grew 17.8-fold (n = 7) on average, while tumors treated at the indicated periods with low-dose or high dose FTS grew 15.8-fold (n = 7) and 7.7-fold (n = 5), respectively. Compared to the average tumor size in the control group, tumor size after 3 weeks in the low-dose and high-dose FTS-treated groups were 88.6% (not significant) and 50.7% (P = 0.019), respectively.

FTS inhibits mammary tumor growth. Next, we examined the effect of Ras inhibition on mammary tumor growth. The effect of FTS treatment on tumor growth is depicted in Figure 3. Over the 3 weeks of the experiment, tumors in the control mice (treated with the vehicle alone 0.5% CMC) grew 17.8 fold, while tumors treated with 10 mg/kg FTS grew 15.8 fold (to 88.6% of tumor volume in the control group), and tumors treated with 40 mg/kg FTS grew 7.7 fold (to 50.7% of tumor volume in the control group; n = 12; P < 0.02) (Fig. 3). These results demonstrated that FTS treatment inhibits the growth of mammary tumors.

### *In-vivo* effect of FTS on Ras signaling

**Fig. 4:**
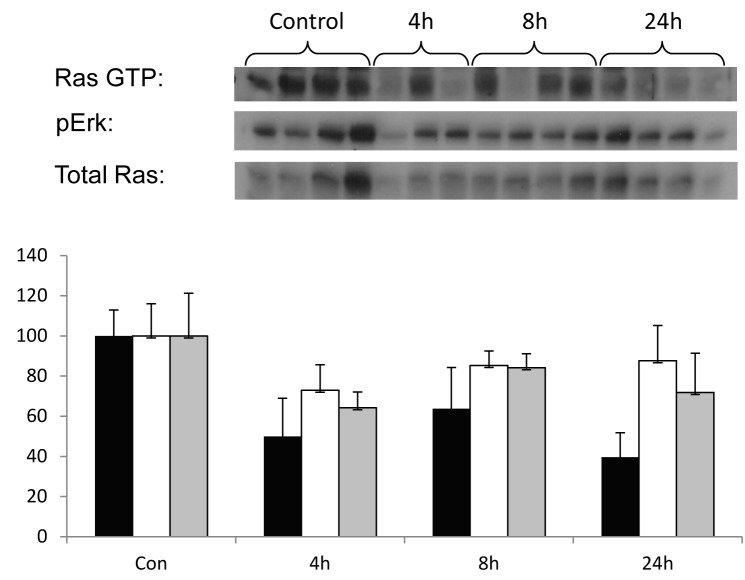
Inhibition of Ras in tumors affects Ras and pERK levels after a single dose of FTS (A), Activated Ras (Ras-GTP), pERK, and total Ras were assayed in xenografts prepared from DA3 tumors removed from mice at the indicated time periods after treatment with FTS (40 mg/kg) or vehicle (0.5% CMC). Western blot analysis demonstrates the rapid effect of FTS on ERK phosphorylation and on Ras activation and expression *in vivo*. (B), Bar graph depicting the average intensity (%) of each treatment relative to the control group. Black Bars represent Ras-GTP, white Bars represent pERK, and gray Bars represent total Ras. The results showed that FTS inhibits Ras-GTP, pERK, and total Ras as early as 4 hours after treatment.

The effect of FTS on Ras signaling was measured at the specified time points in tumors from mice treated with 40 mg/kg FTS. Western blot analysis (Fig. 4A) shows the effects of FTS on Ras-GTP, pERK, and total Ras in the tumors of mice sacrificed 4 hours (lanes 5 to 7), 8 hours (lanes 8 to 11), or 24 hours (lanes 12 to15) after FTS treatment compared to those of control mice treated with the vehicle alone (lanes 1 to 4). These results showed that Ras signaling in tumors mammary is inhibited from as early as 4 hours after treatment with FTS.

### Inhibition of Ras prevents the HGF/SF-induced increase in tumor blood volume

**Fig. 5:**
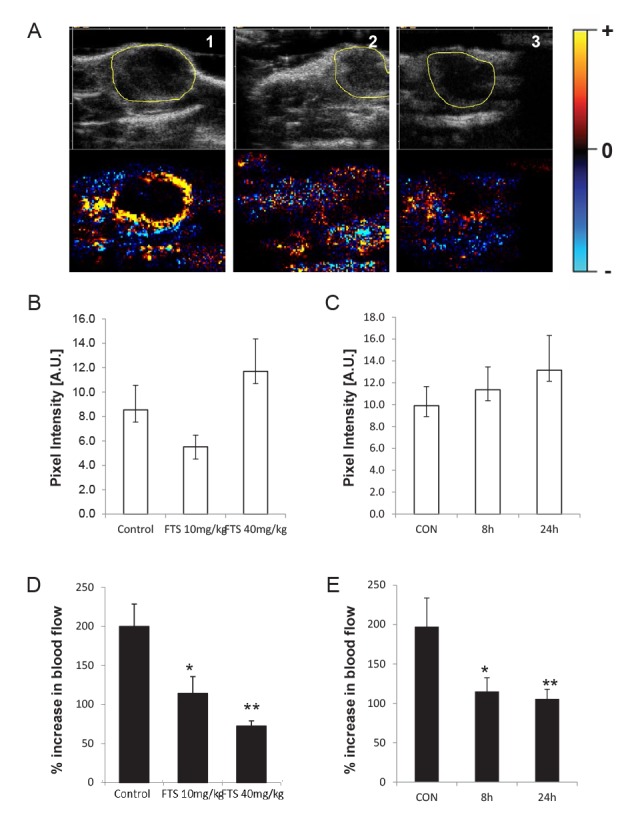
Effects of Ras on HGF/SF-induced changes in tumor blood volume Mice bearing DA3 mammary adenocarcinoma tumors were treated either with vehicle alone (CMC 5%) or with FTS. Tumor blood volume was measured by CM ultrasound (As described in materials and methods) and the effect Ras inhibition on HGF/SF-induced blood flow increase was calculated. (A), an example of the effect of FTS on HGF/SF-induced hemodynamic changes. Tumor outlines were marked (top) and the changes in blood volume in the tumors were calculated. Maps depicting the change in tumor blood flow after HGF/SF administration compared to the baseline blood flow were generated (bottom). Yellow pixels depict increased blood volume after HGF/SF injection: black, no change; blue; decrease. (A1), vehicle-treated tumors showed an increase in blood volume after HGF/SF administration. Tumors treated (A2) with 10 mg/kg FTS or (A3) with 40 mg/kg FTS did not show this characteristic increase in blood volume. Quantitative analysis of basal tumor blood volume shows that it was not affected by either (B) long-term (5 times a week for 3 weeks, as described in materials and methods) or (C) short-term (single administration FTS) treatment with FTS (n = 33). In contrast, when the effect of FTS on HGF/SF-induced tumor blood volume was examined we demonstrate that vehicle treated mice displayed a 2 fold increase in tumor blood volume both in long and short term (D) treatments while long-term (E) treatment with either 10 mg/kg FTS (n = 18) or 40 mg/kg FTS (n = 21); P = 0.036 or P = 0.0002, respectively) (D), as well as by short-term FTS treatment, which was manifested as early as 8 hours after treatment was started (by 42%, n = 28, P = 0.035) (E) that was apparent also in mice treated with FTS 24 hours prior to imaging(47%, n=26, P = 0.028) (E).

We next examined the hemodynamic effects of Ras inhibition. Mice bearing DA3 mammary xenograft tumors (average volume, 10 mm3) were treated 5 times a week for 3 weeks with 40 mg/kg (high dose) or 10 mg/kg (low dose) of FTS dissolved in 0.5% CMC. Tumor blood volume was measured by US-CMI as described in Materials and Methods. In a previous study we showed that HGF/SF treatment *in vivo* leads to an increase of 200% to 300% in tumor blood volume [13]. In the present study we examined whether this phenomenon is abolished by the Ras inhibitor FTS. Treatment with FTS alone induced a slight, nonsignificant change in basal tumor blood flow (Fig. 5A). In mice treated with either low-dose (n = 18) or high-dose (n = 21) FTS, the HGF/SF-induced increase in tumor blood volume was significantly inhibited (P = 0.036;,P = 0.0002, respectively) (Fig. 5C). These results, by demonstrating that FTS inhibits the effect induced by HGF/SF on tumor blood volume, show that this induction is mediated through the Ras signaling cascade. Moreover, the higher dose of FTS used in this study led to the inhibition of blood volume in the tumor following HGF/SF administration, demonstrating the potency of the inhibitor.

### Time dependency of the inhibitory effect of Ras on the HGF/SF-induced increase in tumor blood volume

The inhibitory effects of FTS (40 mg/kg) on basal (vehicle-treated) and on HGF/SF-induced tumor blood volumes were evaluated at different times between 2 hours and 24 hours after treatment. Comparison of tumor blood volume in vehicle-treated mice before and after treatment with FTS alone showed that within this time frame blood volume was not affected by FTS (Fig. 5) (n = 47). In contrast, FTS treatment significantly inhibited HGF/SF-induced increase in blood volume (n = 37, P (Anova) = 0.015). The inhibition was observed as early as 8 hours after FTS treatment (n = 28, P = 0.035) and after 24 hours the observed decrease in HGF/SF-induced tumor blood volume was also significant (n = 26, P = 0.028) (Fig. 5B).

## DISCUSSION

Intensive research has been devoted over the last decades to elucidating the signaling pathway leading from HGF/SF to Met phosphorylation and Ras activation and to the biological outcomes of this signaling pathway. However, the metabolic effects of HGF/SF-Met and Ras signaling *in vitro*, as well as *in vivo*, are poorly understood. The aim of this study was to gain a better understanding of the interplay between HGF/SF-Met–Ras signaling, tumor metabolism, and blood flow. Our results *in vitro* showed that FTS inhibits HGF/SF-induced Ras activity, Erk phosphorylation, and cell motility. In addition, basal cell motility was inhibited by FTS. These results support published data by our group demonstrating that FTS inhibits Ras signaling and cell motility in a variety of tumor cell lines [29]. The basal inhibition of signaling and metabolism by FTS may be explained by the constitutive activation of Met in our *in-vitro* model [35] and further demonstrates the importance of Ras participation in Met signaling and in mechanisms of cell motility.

In agreement with our previously published data was the present finding that HGF/SF induced an increase in glucose uptake in our *in vitro* model of DA3 cells [11]. However, Met, and subsequently, Ras activation by HGF/SF did not lead to a significant increase in ATP. However, inhibition of Ras increased glucose uptake but decreased the steady-state level of ATP in these cells. Combined treatment with FTS and HGF/SF led to an additive increase in glucose uptake, while ATP levels returned to basal (results are summarized in table 1). These findings indicated that inhibition of Ras leads to decoupling between glucose uptake and ATP synthesis. Ras was previously shown to control tumor metabolism by stimulating glucose uptake and channeling glucose intermediates into the hexosamine biosynthesis and pentose phosphate pathways [17]. In a previous study we showed that FTS-induced inhibition of Ras signaling induces increased glucose uptake in non-malignant cells as well [22]. The present finding that Ras increased glucose uptake but inhibited ATP synthesis demonstrates a mismatch between glucose metabolism and ATP synthesis. However, HGF/SF overcame the observed effect of FTS on ATP steady state. We therefore suggest that HGF/SF can restore the physiological link between glucose consumption and ATP production, possibly through the mediation of metabolic pathways triggered by signaling pathways other than Ras.

**Table 1:** A summary of the effect of Met activation by HGF/SF, Ras inhibition by FTS and the combination of the two treatments on cell motility, metabolism and tumor blood volume

	HGF/SF	FTS	HGF/SF + FTS
**Motility**	↑↑↑	↓↓	↑
**Glucose uptake**	↑	↑	↑↑
**ATP**	-	↓↓	-
**Tumor Blood volume**	↑↑↑	-	↓

To examine the effects of interplay between Ras inhibition and HGF/SF-Met *in vivo*, we measured their effects on tumor growth and blood flow. Our finding that Ras inhibition hindered tumor growth is in accord with other studies, in which doses similar to those used in the present study inhibited the growth of breast cancer [34] as well as of other malignancies such as pancreatic tumors [31, 36], glioblastomas [33], and lung cancer [37].

Blood flow and metabolism are interconnected. In normal tissues, increased metabolism leads to hypoxia and hypoglycemia, with consequent increases in blood flow [38], which leads in turn to increased metabolite availability [39]. Blood flow and metabolism have been shown to be mismatched in some tumors [2]. The mismatch, and in particular high metabolism relative to low blood flow, can be detected in tumors by functional and molecular imaging**,** and is associated with poor response to treatment and with early relapse or disease progression [2]. Studies in our laboratory, using NMR and oxygen measurements *in vitro* and fMRI *in vivo*, have shown that activation of the Met tyrosine kinase receptor leads to increased consumption of glucose and oxygen [11, 14]. We hypothesize that the resulting hypoxia and hypoglycemia lead to the changes that we reported in tumor blood flow *in vivo* [13, 14].

The molecular mechanism underlying the hemodynamic changes induced by HGF/SF is still unclear. Here we examined the effect of Met inhibition downstream of the Ras signaling junction on tumor blood flow, and on the interplay between signaling, metabolism and blood flow. Our results demonstrated that HGF/SF increases blood flow in DA3 tumors. Ras inhibition by FTS reduced the ATP steady state but FTS treatment did not by itself affect tumor blood volume; however, it abolished the HGF/SF-induced increase in tumor blood volume, and it did this despite the increase in glucose uptake shown here to be induced by FTS.

Our results further demonstrated that Ras inhibition is apparent as early as 4 hours after administration of FTS. We also showed that Met activation induces an increase in tumor blood flow as early as 20 minutes after HGF/SF administration, pointing to recoupling of metabolism and blood flow. Inhibition of Met via Ras inhibition takes effect *in vivo*, restoring the decoupling, as soon as 8 hours after FTS administration. This demonstrates the dynamic nature of metabolic alterations and hemodynamics in the tumor.

In this study we used our previously established Met functional molecular imaging (Met-FMI) technique to evaluate the effect of Ras inhibition on hemodynamic changes induced by HGF/SF [13]. Using Met-FMI we were able to detect inhibition of Met signaling by FTS in a manner of hours after Ras administration. We also showed that the inhibition of Ras by FTS may be used for anti-Met targeted therapy. Better understanding of the fragile interplay between signaling, metabolism, and blood flow in malignant tissues may shed light on tumor progression and could potentially be used in the future to develop functional imaging modalities for prescribing and evaluating personalized anti-Met and anti-Ras targeted therapy.

## MATERIALS AND METHODS

### Cell line

The mouse mammary adenocarcinoma cell line, D1-DMBA-3 (DA3), induced in female BALB/c mice by dimethylbenzanthracene [40], kindly provided by Diana M. Lopez, Sylvester Comprehensive Cancer Center for the State of Florida, Miami, Florida. Cell authentication was confirmed most recently by cDNA array at 2012.

### S-trans,trans-farnesylthiosalicylic acid (FTS)

Western blot analysis. DA3 cells were incubated for 24 h in medium containing 10% fetal calf serum (FCS) and 50 to 100 μM FTS or 0.1% DMSO. The cells were lysed with solubilization buffer (50 mmol/L Tris-HCl (pH 7.6), 20 mmol/L MgCl_2_, 200 mmol/L NaCl, 0.5% NP40, 1 mmol/L DTT, and protease inhibitors). Lysates were used to determine the Ras-GTP content of the cells by the glutathione S-transferase – Ras-binding domain pull-down assay, followed by Western immunoblotting with mouse anti pan-Ras antibody (1:2500). Total Ras proteins, phospho-ERK (pERK), and β-tubulin (control) in the cell lysates were also determined by immunoblotting, using the above anti-Ras antibodies, rabbit anti-phospho-ERK1/2 antibody (1:10,000; Sigma-Aldrich, St. Louis, MO), and rabbit anti-β-tubulin (1:1000; Santa Cruz Biotechnology, Santa Cruz, CA). Immunoblots were exposed to either peroxidase-goat anti-mouse IgG or peroxidase-goat anti-rabbit IgG (1:2500). Protein bands were visualized by enhanced chemiluminescence and quantified with ImageJ (developed by W. Rasband at the U.S. National Institutes of Health and available at http://rsbweb.nih.gov/ij/).

### Scratch assay

Scratch assay was carried out with DA3 cells in 24-well plates. Cells were treated with FTS (5 μM) or only with vehicle (0.005% DMSO in DMEM containing 0.1% FCS) (control) for 4 hours. A 200-μl sterile tip was used to inflict a scratch in each well and HGF/SF was added to half of the wells. The four treatments were: (1) Non- treated (control), (2) HGF/SF, (3) FTS and (4) HGF/SF + FTS. Cells were imaged, 5 hours and 13 hours after the scratch, by time-lapse confocal laser scanning microscopy (CLSM-510, Zeiss, Germany). The scratch was measured (in mm2) at both time points using ImageJ, and the motility rate of cells the wound was calculated.

### Imaging of glucose uptake

Cells were grown on glass bottom plates (CELLviewTM, Greiner Bio-One GmbH, Germany) overnight. The cells were starved of serum (control) or were treated with HGF/SF in starvation medium (starvation medium - containing 0.1% FCS and 5 mM glucose), for 24 hours. Four hours prior to imaging FTS (5 μM) was added to half of the plates. The four treatment groups: (1) non-treated, (2) treated with HGF/SF, (3) treated with FTS and (3) treated with HGF/SF + FTS. They were then incubated for 30 minutes at 37°C in culture medium containing 100 μM fluorescent 2-deoxy-2-[(7-nitro-2,1,3-benzoxadiazol-4-yl)amino]-D-glucose (2-NBDG) (Molecular Probes–Invitrogen, Carlsbad, CA). The cells were then washed thoroughly to remove the 2-NDBG-containing medium and were imaged using a Leica SP5 microscope (Leica, Heidelberg, Germany). Image analysis was performed using ImageJ.

### Animal model

All experiments with animal models were done in compliance with the principles of the National Research Council (NRC) and were approved by the institutional animal care and use committee (IACUC) (#M-09-005).

To induce xenograft tumors in mice, DA3 cells [40] were injected into the lower left mammary gland of 8 week old (17-22 gr) female BALB/c mice (5 × 10^5^ cells in 100 μL saline). Tumor dimensions were measured by ultrasound imaging. Volume was calculated by the formula length × width × depth / 2. When tumors reached 10 mm3, FTS treatment was started.

To examine the effects of FTS inhibition on tumor growth, mice bearing DA3 tumors were fed with FTS dissolved in 0.5% carboxymethycellulose (CMC) at two concentrations, high dose (HD) 40 mg/kg or low dose (LD) 10 mg/kg, 5 times a week for 3 weeks after tumor appearance. Tumor dimensions were measured weekly by ultrasound.

### Met functional molecular imaging using contrast medium-enhanced ultrasound imaging

Measurement by US-CMI was carried out as previously described [13]. In short, mice were anesthetized by inhalation of 2% isoflurane (Halocarbon Products, River Edge, NJ) delivered with oxygen, using a non-rebreathing anesthetic delivery system (Summit Anesthesia Solutions, Bend, OR). Anesthetized mice were placed on a heating pad to maintain body temperature and minimize temperature-induced changes in blood flow. Prior to imaging, hair surrounding the tumor area was removed with depilatory cream. Imaging gel was spread over the tumor, and a 27-gauge needle was inserted into the tail vein to allow repeated intravenous (i.v.) injections. Following i.v. injection of 15 μg of Definity (Perflutren Lipid Microsphere), dynamic non-destructive US-CMI was performed in contrast-media specific mode. Ultrasound measurements were taken during microbubble injection at 20 frames/sec with a fixed 15L8s, 14-MHz linear transducer power (18 dB/0.25 MI) (Acuson Sequoia 512™, Mountain View, CA). To enable repeated imaging after HGF/SF injection (1000 units of purified human HGF/SF for each mouse), microbubbles were destroyed by destructive mode imaging. For Met functional molecular imaging (Met-FMI), the effect of HGF/SF on tumor maximum peak enhancement was calculated by US-CMI before and 20 min after i.v. HGF/SF-induced activation of Met.

Imaging settings were standardized, and were unchanged throughout the experiment. No major near-field artifacts were encountered.

### Image analysis

Contrast media (CM) enhancement was calculated from time series images (clips) using dedicated FMI analysis software, UIA (I-Labs, Petah Tikva, Israel). Tumor ROI (region of interest) was determined on the B-mode ultrasound image. CM enhancement was calculated as the difference between the maximal signal intensity after CM injection and the background (intensity of the ROI before CM injection) [13].

Average signal intensity curves were calculated by fitting to an exponential curve: Y = C – A(1 – e–βt), where C represents background noise, A represents volume, t represents time, and β represents flow. HGF/SF-induced changes in maximum peak enhancement were calculated as the post-HGF/SF CM enhancement divided by the pre-HGF/SF CM enhancement. Statistical analysis was performed using Student's t test (with Microsoft Excel). The effect of HGF/SF was mapped by comparison of the CM-enhancement maps generated for pre-HGF/SF (saline) with post-HGF/SF treatments for each mouse. Maps of the HGF/SF tumor enhancement effect demonstrate changes in contrast enhancement upon HGF/SF treatment (post-map minus pre-map values).

## References

[1] Jain RK (2005). Antiangiogenic therapy for cancer: current and emerging concepts. Oncology (Williston Park).

[2] Mankoff DA, Dunnwald LK, Partridge SC, Specht JM (2009). Blood flow-metabolism mismatch: good for the tumor, bad for the patient. Clin Cancer Res.

[3] Gherardi E, Birchmeier W, Birchmeier C, Vande Woude G (2012). Targeting MET in cancer: rationale and progress. Nat Rev Cancer.

[4] Birchmeier C, Birchmeier W, Gherardi E, Vande Woude GF (2003). Met, metastasis, motility and more. Nat Rev Mol Cell Biol.

[5] Gibney GT, Aziz SA, Camp RL, Conrad P, Schwartz BE, Chen CR, Kelly WK, Kluger HM c-Met is a prognostic marker and potential therapeutic target in clear cell renal cell carcinoma. Ann Oncol.

[6] Goyal L, Muzumdar MD, Zhu AX Targeting the HGF/c-MET Pathway in Hepatocellular Carcinoma. Clin Cancer Res.

[7] Ponzo MG, Lesurf R, Petkiewicz S, O'Malley FP, Pinnaduwage D, Andrulis IL, Bull SB, Chughtai N, Zuo D, Souleimanova M, Germain D, Omeroglu A, Cardiff RD, Hallett M, Park M (2009). Met induces mammary tumors with diverse histologies and is associated with poor outcome and human basal breast cancer. Proc Natl Acad Sci U S A.

[8] Kaposi-Novak P, Lee JS, Gomez-Quiroz L, Coulouarn C, Factor VM, Thorgeirsson SS (2006). Met-regulated expression signature defines a subset of human hepatocellular carcinomas with poor prognosis and aggressive phenotype. J Clin Invest.

[9] Underiner TL, Herbertz T, Miknyoczki SJ (2010). Discovery of small molecule c-Met inhibitors: Evolution and profiles of clinical candidates. Anticancer Agents Med Chem.

[10] Rodig SJ, Shapiro GI Crizotinib, a small-molecule dual inhibitor of the c-Met and ALK receptor tyrosine kinases. Curr Opin Investig Drugs.

[11] Kaplan O, Firon M, Vivi A, Navon G, Tsarfaty I (2000). HGF/SF activates glycolysis and oxidative phosphorylation in DA3 murine mammary cancer cells. Neoplasia.

[12] Pennacchietti S, Michieli P, Galluzzo M, Mazzone M, Giordano S, Comoglio PM (2003). Hypoxia promotes invasive growth by transcriptional activation of the met protooncogene. Cancer cell.

[13] Tsarfaty G, Stein GY, Moshitch-Moshkovitz S, Kaufman DW, Cao B, Resau JH, Vande Woude GF, Tsarfaty I (2006). HGF/SF increases tumor blood volume: a novel tool for the in vivo functional molecular imaging of Met. Neoplasia.

[14] Shaharabany M, Abramovitch R, Kushnir T, Tsarfaty G, Ravid-Megido M, Horev J, Ron R, Itzchak Y, Tsarfaty I (2001). In vivo molecular imaging of met tyrosine kinase growth factor receptor activity in normal organs and breast tumors. Cancer research.

[15] Gao CF, Vande Woude GF (2005). HGF/SF-Met signaling in tumor progression. Cell Res.

[16] Gaglio D, Metallo CM, Gameiro PA, Hiller K, Danna LS, Balestrieri C, Alberghina L, Stephanopoulos G, Chiaradonna F (2011). Oncogenic K-Ras decouples glucose and glutamine metabolism to support cancer cell growth. Mol Syst Biol.

[17] Ying H, Kimmelman AC, Lyssiotis CA, Hua S, Chu GC, Fletcher-Sananikone E, Locasale JW, Son J, Zhang H, Coloff JL, Yan H, Wang W, Chen S, Viale A, Zheng H, Paik JH Oncogenic Kras maintains pancreatic tumors through regulation of anabolic glucose metabolism. Cell.

[18] Chapman MS, Miner JN (2011). Novel mitogen-activated protein kinase kinase inhibitors. Expert Opin Investig Drugs.

[19] Pao W, Miller V, Zakowski M, Doherty J, Politi K, Sarkaria I, Singh B, Heelan R, Rusch V, Fulton L, Mardis E, Kupfer D, Wilson R, Kris M, Varmus H (2004). EGF receptor gene mutations are common in lung cancers from “never smokers” and are associated with sensitivity of tumors to gefitinib and erlotinib. Proc Natl Acad Sci U S A.

[20] Danilkovitch-Miagkova A, Zbar B (2002). Dysregulation of Met receptor tyrosine kinase activity in invasive tumors. J Clin Invest.

[21] Deberardinis RJ, Sayed N, Ditsworth D, Thompson CB (2008). Brick by brick: metabolism and tumor cell growth. Current opinion in genetics & development.

[22] Mor A, Aizman E, George J, Kloog Y Ras inhibition induces insulin sensitivity and glucose uptake. PLoS One.

[23] Aharonson Z, Gana-Weisz M, Varsano T, Haklai R, Marciano D, Kloog Y (1998). Stringent structural requirements for anti-Ras activity of S-prenyl analogues. Biochim Biophys Acta.

[24] Kloog Y, Cox AD (2004). Prenyl-binding domains: potential targets for Ras inhibitors and anti-cancer drugs. Semin Cancer Biol.

[25] Kloog Y, Cox AD (2000). RAS inhibitors: potential for cancer therapeutics. Mol Med Today.

[26] Blum R, Kloog Y (2005). Tailoring Ras-pathway--inhibitor combinations for cancer therapy. Drug Resist Updat.

[27] Yaari S, Jacob-Hirsch J, Amariglio N, Haklai R, Rechavi G, Kloog Y (2005). Disruption of cooperation between Ras and MycN in human neuroblastoma cells promotes growth arrest. Clin Cancer Res.

[28] Blum R, Jacob-Hirsch J, Amariglio N, Rechavi G, Kloog Y (2005). Ras inhibition in glioblastoma down-regulates hypoxia-inducible factor-1alpha, causing glycolysis shutdown and cell death. Cancer research.

[29] Goldberg L, Kloog Y (2006). A Ras inhibitor tilts the balance between Rac and Rho and blocks phosphatidylinositol 3-kinase-dependent glioblastoma cell migration. Cancer research.

[30] Jansen B, Schlagbauer-Wadl H, Kahr H, Heere-Ress E, Mayer BX, Eichler H, Pehamberger H, Gana-Weisz M, Ben-David E, Kloog Y, Wolff K (1999). Novel Ras antagonist blocks human melanoma growth. Proc Natl Acad Sci U S A.

[31] Goldberg L, Israeli R, Kloog Y (2012). FTS and 2-DG induce pancreatic cancer cell death and tumor shrinkage in mice. Cell Death Dis.

[32] Laheru D, Shah P, Rajeshkumar NV, McAllister F, Taylor G, Goldsweig H, Le DT, Donehower R, Jimeno A, Linden S, Zhao M, Song D, Rudek MA, Hidalgo M (2012). Integrated preclinical and clinical development of S-trans, trans-Farnesylthiosalicylic Acid (FTS, Salirasib) in pancreatic cancer. Investigational new drugs.

[33] Aizman E, Mor A, Levy A, George J, Kloog Y (2012). Ras inhibition by FTS attenuates brain tumor growth in mice by direct antitumor activity and enhanced reactivity of cytotoxic lymphocytes. Oncotarget.

[34] Santen RJ, Lynch AR, Neal LR, McPherson RA, Yue W (2006). Farnesylthiosalicylic acid: inhibition of proliferation and enhancement of apoptosis of hormone-dependent breast cancer cells. Anticancer Drugs.

[35] Firon M, Shaharabany M, Altstock RT, Horev J, Abramovici A, Resau JH, Vande Woude GF, Tsarfaty I (2000). Dominant negative Met reduces tumorigenicity-metastasis and increases tubule formation in mammary cells. Oncogene.

[36] Haklai R, Elad-Sfadia G, Egozi Y, Kloog Y (2008). Orally administered FTS (salirasib) inhibits human pancreatic tumor growth in nude mice. Cancer Chemother Pharmacol.

[37] Zundelevich A, Elad-Sfadia G, Haklai R, Kloog Y (2007). Suppression of lung cancer tumor growth in a nude mouse model by the Ras inhibitor salirasib (farnesylthiosalicylic acid). Mol Cancer Ther.

[38] Feigl EO (2004). Berne's adenosine hypothesis of coronary blood flow control. Am J Physiol Heart Circ Physiol.

[39] Song CW, Park HJ, Lee CK, Griffin R (2005). Implications of increased tumor blood flow and oxygenation caused by mild temperature hyperthermia in tumor treatment. Int J Hyperthermia.

[40] Fu Y, Watson G, Jimenez JJ, Wang Y, Lopez DM (1990). Expansion of Immunoregulatory Macrophages by Granulocyte-Macrophage Colony-stimulating Factor Derived from a Murine Mammary Tumor. Cancer Res.

